# Simultaneous Integrated Boost Intensity-Modulated Radiation Therapy Can Benefit the Locally Advanced Rectal Cancer Patients With Clinically Positive Lateral Pelvic Lymph Node

**DOI:** 10.3389/fonc.2020.627572

**Published:** 2021-02-22

**Authors:** Shuai Li, Yangzi Zhang, Yang Yu, Xianggao Zhu, Jianhao Geng, Huajing Teng, Zhilong Wang, Tingting Sun, Lin Wang, Hongzhi Wang, Yongheng Li, Aiwen Wu, Yong Cai, Weihu Wang

**Affiliations:** ^1^ Department of Radiation Oncology, Key Laboratory of Carcinogenesis and Translational Research (Ministry of Education/Beijing), Peking University Cancer Hospital and Institute, Beijing, China; ^2^ Department of Gastrointestinal Surgery, Key laboratory of Carcinogenesis and Translational Research (Ministry of Education), Peking University Cancer Hospital & Institute, Beijing, China; ^3^ Department of Radiology, Key laboratory of Carcinogenesis and Translational Research (Ministry of Education), Peking University Cancer Hospital & Institute, Beijing, China

**Keywords:** simultaneous integrated boost intensity-modulated radiation therapy, neoadjuvant chemoradiotherapy, lateral pelvic lymph node, local advanced rectal cancer, regrowth rate, disease-free survival

## Abstract

**Background and Purpose:**

The optimal treatment modality for clinically positive lateral pelvic lymph node (LPLN) from locally advanced rectal cancer (LARC) is unknown. Thus, we aimed to analyze the optimal radiotherapy dose for clinically positive LPLN from LARC.

**Materials and Methods:**

We retrospectively evaluated distal LARC (i.e., within 8 cm from the anal verge) patients with clinically positive LPLN (i.e., ≥7 mm in the short axis). They were divided into two groups based on whether or not they received simultaneous integrated boost intensity-modulated radiation therapy (SIB-IMRT)–based chemoradiotherapy. The total radiotherapy dose on LPLN were 56-60Gy for SIB-IMRT group and 41.8Gy for non-SIB-IMRT group. The clinical parameters and regrowth rate of LPLN were then compared between the two groups.

**Results:**

A total of 151 patients were evaluated, and 83 and 68 patients were classified to the SIB-IMRT and non-SIB-IMRT group, respectively. The median follow-up period was 22.6 months, and the 2-year LPLN regrowth rate was significantly different between the SIB-IMRT group and the non-SIB-IMRT group (0% vs 10.8%, P=0.024). Further, SIB-IMRT yielded a significantly lower 2-year LPLN regrowth rate in patients whose LPLN measured ≥8 mm in the short axis (0% vs. 15.9%, P=0.019) or ≥10 mm in the long axis (0% vs. 17.6%, P=0.024) compared to patients who were in non-SIB-IMRT group. Meanwhile, there was no significant difference in grade II radiation-related toxicity (30.1% vs. 39.1%, P=0.217) and surgical complications (21.8% vs. 12.2%, P=0.198) between the two groups.

**Conclusion:**

SIB-IMRT–based neoadjuvant chemoradiotherapy is beneficial for eliminating clinically positive LPLN from LARC without increasing the incidence of radiotherapy-related toxicity and surgical complications, and patients with larger LPLN may gain benefit from this technique.

## Introduction

Involvement of the lateral pelvic lymph node (LPLN) occurs in 7%–15% of cases of locally advanced rectal cancer (LARC) and is particularly more frequent in those with cT3-4 or distal disease (1–4). The Japanese Society for Cancer of the Colon and Rectum recommends lateral pelvic lymph node dissection (LPLD) for these patients (5). However, LPLD involves a long operation time and may have adverse effects on urinary and male sexual function (6–8). One study compared the patterns of local recurrence between neoadjuvant chemoradiotherapy (NCRT) and LPLD for LARC, and found similar recurrence rates (9). LPLD may be an overtreatment for negative LPLN patients.

Although magnetic resonance imaging (MRI) has been recommended as the standard radiology modality for evaluating LARC (10), there is still no consensus on the optimal method for determining the status of lateral lymph node metastasis. Ogawa et al. reported that a 5-mm short axis cutoff of LPLN had nearly 80% accuracy for diagnosing positive LPLN (11). In addition to the issues in diagnosis, the optimal treatment modality for positive LPLN remains unclear. Although comprehensive treatment for distal LARC includes NCRT and total mesorectal excision (TME) (10, 12), some patients still develop lateral pelvic recurrence (13–15). Akiyoshi et al. found that LPLNs with a short-axis diameter of ≥8 mm were associated with a higher metastasis rate even after NCRT (16). Atsushi et al. also indicated that a 7-mm short axis may be a risk factor for lateral local recurrence (15). Collectively, these results suggest that standard NCRT may be an inefficient treatment for large LPLN.

Radiation dose escalation could be a non-surgical strategy for improving the outcomes of local treatment for large LPLN. The simultaneous integrated boost (SIB) technique, which involves providing corresponding doses to different target areas, has been widely used in lung cancer and some abdominal cancers. In our center, SIB-IMRT has been used as a standard NCRT technique in rectal cancer (17). From 2016, some LARC patients with suspected positive LPLN underwent SIB-IMRT on the LPLNs plus a suitable margin to improve the local effect in our institution. This study aimed to analyze the safety and effectiveness of SIB-IMRT for clinically positive LPLN from distal LARC to ultimately determine the optimal radiotherapy dose.

## Materials and Methods

### Study Design and Patients

This was a retrospective study of rectal cancer patients who underwent neoadjuvant chemoradiotherapy (NCRT) in our center between January 2016 and June 2019. The inclusion criteria were as follows: (1) pathologically confirmed rectal adenocarcinoma, (2) tumor location within 8 cm from the anal verge, (3) clinical suspicion of LPLN on MRI, (4) age from 18 to 75 years, (5) an Eastern Cooperative Oncology Group performance status of 0–1, (6) no other primary malignancies or life-threatening vascular diseases, and (7) good compliance to the whole treatment and follow-up. Data were collected from the medical records. The patients were divided into two groups based on whether or not they received SIB-IMRT-based chemoradiotherapy.

This study was approved by the institutional review board of Ethics Committee of Beijing Cancer Hospital (approval number: 2020YJZ71) and was conducted according to the Declaration of Helsinki. Written informed consent was obtained after informing each patient about the possible risks and benefits.

### MRI Assessment

Pretreatment MRI involved T2-weighted thin-section sequences in oblique axial planes, with the oblique axial scans perpendicular to the long axis of the rectum (3-mm slices) (18). MR images were read by one radiologist and one radiation oncologist. Clinically positive LPLN was defined as a short axis of ≥7mm, adding regular margin or mix signals. A clinically positive diagnosis was achieved by consensus between the radiologist and the radiation oncologist. Data regarding mesorectal lymph node metastases, extramural depth of tumor invasion, external mesorectal vascular invasion, and mesorectal fascia (MRF) status were also recorded (19). After NCRT, MRI was performed again to reassess the short axis of LPLN, tumor regression grade, MRF status, and T/N stage. For patients who did not undergo surgery, MRI was used as routine radiology modality to assess early regrowth.

### Treatment

#### Neoadjuvant Chemoradiotherapy

Enhanced computed tomography (CT) simulation was recommended for all patients. A full bladder was required to protect the intestine. More than 90% of patients were positioned supine to reduce axial displacement, and a thermoplastic film and abdominal plate board were used to fix the position. The MRI simulation with the same position and condition was applied as a reference to ensure accurate target contour. The IMRT technique was used for all patients; the details of the target contour and the prescribed dose have been described previously (17). Total radiation doses of 50.6 and 41.8 Gy were delivered to the planning gross target volume (PGTVp) and planning target volume (PTV), respectively. For partial patients, the gross target volume of the positive lymph nodes (GTVn) was used to delineate the large LPLNs, while the PGTVn was a 5-mm extension of the GTVn in three dimensions. The dose-fractionation schemes for PGTVn were prescribed by the treating physicians. The SIB-IMRT technique was delivered to the PGTVn at 2.54-2.72 Gy per fraction for a total dose of 56-60 Gy. For other patients, the LPLN was in the PTV coverage.

Synchronous chemotherapy was individualized. Oral capecitabine was administered at 825 mg/m^2^ twice daily for 5 days/week (17). Patients with high-risk factors (e.g., MRF positivity or extramural vascular invasion (EMVI)) were recommended for additional oxaliplatin that was administered at 85 mg/m^2^ every 2 weeks or 50 mg/m^2^ weekly.

#### Chemotherapy

Since the introduction of total neoadjuvant therapy as a new treatment paradigm for LARC (20), induction or consolidation chemotherapy has been used to improve tumor regression and the probability for anal sphincter preservation. Chemotherapy comprised oral capecitabine (1,000 mg/m^2^ twice daily, d1-14/Q21d) and CapOX (oxaliplatin 130 mg/m^2^, d1; capecitabine 1,000 mg/m^2^ twice daily, d1-14/Q21d). Adjuvant CapeOX chemotherapy was recommended for every patient, and FOLFOX or capecitabine monotherapy were considered alternatives.

#### Surgery

Surgery was recommended after NCRT and adequate radiology evaluation following the TME criteria. The surgical strategies included low anterior resection, abdominoperineal resection, Hartmann and trans-anal local resection. LPLD was performed based on the radiologic findings of positive LPLN and the surgeons’ decision. The surgical TME specimen was assessed for the T and N stages and for pathologic CRM status (defined as a tumor present within ≤1 mm from the radial resection margin).

The tumor regression grade was assessed according to the NCCN criteria (21). R0 resection was defined as the tumor being more than 1 mm from the circumferential resection margin or other positive margins. Patients with a clinical complete response (cCR) or those with a strong desire to preserve the anus did not undergo surgery but were placed on need intensive follow-up. Follow-up included digital examination, radiology evaluation, serum carcinoembryonic antigen, and colonoscopy.

### Treatment Outcomes and Outcome Measures

NCRT-related acute toxicity was graded by a radiation oncologist through weekly outpatient evaluations following the Common Terminology Criteria of Adverse Events Version 4.0. Postoperative complications were also recorded. The patients were followed up every 3 months for 2 years after treatment, then every 6 months for the next 3 years.

The primary outcome was LPLN regrowth rate, and the LPLN regrowth was defined as any one of the three situations below: the progress response of LPLN, or the regrowth of LPLN which obtain complete or partial response after NCRT, or newly occurring LPLN in the tumor bed of the LPLD area. The secondary outcome measures were disease-free survival (DFS), treatment toxicity, and surgical complications. The DFS measured from the date of diagnosis to any type of recurrence event. The regrowth of LPLN, locoregional recurrence, distant metastases, and death from any reason were all defined as recurrence events.

### Statistical Analyses

The χ2 test was used to compare differences between the two groups. Survival curves were generated using the Kaplan-Meier method and compared using the log-rank test. All statistical analyses were performed using the Statistical Package for the Social Sciences (IBM Corp., SPSS Statistics for Windows, v. 22.0. (Armonk, NY, USA). P<0.05 was considered statistically significant.

## Results

### Patient Characteristics

In total, 151 LARC patients diagnosed with clinically positive LPLN were included in this study. Among them, 68 patients who received a total dose of 41.8 Gy in 22 fractions on LPLN area were categorized to the non-SIB-IMRT group, and 83 patients who underwent dose escalation *via* the SIB-IMRT technique on LPLN area were classified to the SIB-IMRT group. The median patient age was 57 (range: 20–84) years, and the median distance from the tumor to the anal verge was 4 (range: 0–8) cm. The median short axis and long axis of LPLN was 8 (range: 7–21) mm and 10 (range: 7–30) mm. There were 54 patients (35.8%) with stage T4 disease, 81 patients (53.6%) who were MRF positive, and 75 patients (49.7%) who were EMVI positive. The clinical parameters were well balanced between the two groups besides the SIB-IMRT group has higher proportion of additional oxaliplatin. The patients’ characteristics are shown in **Table 1**.

**Table 1:** The clinical parameters and treatments between the two groups.

Characteristics	SIB-IMRT group (*n* = 83)	Non-SIB-IMRT group (*n* = 68)	*P* value
Age (range)	57 (20–83)	58 (31–81)	–
Gender			
Male	58 (69.9%)	45 (66.2%)	0.627
Female	25 (30.1%)	23 (33.8%)	
Pretreatment CEA			
< 5mol/L	36 (43.4%)	34 (50.0%)	0.697
≥ 5 mol/L	41 (49.4%)	29 (42.6%)	
unidentified	6 (7.2%)	5 (7.4%)	
Distance from anal (range)	4 (0–8) cm	5 (0–8) cm	–
Long axis of tumor (range)	4.8 (1.8–15) cm	5 (2.5–10.5) cm	
Clinical T stage			
T2	3(3.6%)	2 (2.9%)	0.279
T3	55 (66.3%)	37 (54.4%)	
T4	25 (30.1%)	29 (42.6%)	
T3 subgroup			
T3a	6 (7.2%)	1 (1.5%)	0.346
T3b	42 (50.6%)	31 (45.6%)	
T3c	7 (8.3%)	5 (7.4%)	
T3d	0	0	
Clinical N stage			
N1	27 (32.5%)	26 (38.2%)	0.465
N2	56 (67.5%)	42 (61.8%)	
Short axis of LPLN (range)	8 (7–20) mm	8 (7–15) mm	–
<8 mm	31(37.3%)	26(38.2%)	0.911
≥8 mm	52(62.7%)	42(61.8%)	
Long axis of LPLN (range)	8 (7–30) mm	10 (7–21) mm	–
<10 mm	36(43.4%)	28(41.2%)	0.786
≥10 mm	47(56.6%)	40(58.8%)	
MRF Status			
Positive	47(56.6%)	34 (50.0%)	0.417
Negative	36 (43.4%)	34 (50.0%)	
EMVI Status			
Positive	39 (47.0%)	36 (52.9%)	0.467
Negative	44 (53.0%)	32 (47.1%)	
Synchronous chemotherapy			
Capecitabine	48 (57.8%)	57 (83.8%)	**0.001**
Capecitabine+Oxaliplatin	35 (42.2%)	11 (16.2%)	
Neoadjuvant chemotherapy			
CapOX	25 (30.1%)	23 (33.8%)	0.498
Capecitabine	26 (31.3%)	25 (36.8%)	
None	32 (38.6%)	20 (29.4%)	

LPLN, lateral pelvic lymph node; CEA, carcinoembryonic antigen; MRF, mesorectal fascia; EMVI, extramural vascular invasion.

In total, 98 patients (64.9%) received induced or consolidated chemotherapy, and 46 patients (30.5%) had received CapOX synchronous chemotherapy, with this regimen being more commonly used in the SIB-IMRT group (42.2% in the SIB-IMRT group vs 16.2% in the non-SIB-IMRT group, P=0.001).

### Treatment and Survival Outcomes

There were 10 patients (6.6%) who developed distant metastasis after NCRT. In total, 12 patients did not undergo surgery due to medical complications (n=3) and to preserving the anus (n=9). Twenty-five patients (16.6%) achieved cCR. Overall, 104 patients underwent surgery, and the median interval between radiotherapy and surgery was 82 days (range, 32–207 days). In the surgery group, LPLD was performed in 12 patients, none of whom found LPLN metastases. The short-term outcomes after NCRT are shown in **Table 2**. Only the rate of LPLD was significantly different between the two groups (18.2% in the SIB-IMRT group vs 4.1% in the non-SIB-IMRT group, P=0.025).

**Table 2:** Details of short outcome between the two groups.

	SIB-IMRT group (*n* = 83)	Non-SIB-IMRTgroup (*n* = 68)	*p* value
Distant metastasis	4(4.8%)	6(8.8%)	0.325
Refuse surgery	6(7.2%)	3(4.4%)	0.467
Clinical Complete Response	18(18.1%)	7(10.3%)	0.061
Without Surgery due to Medical Complications	0	3(4.4%)	0.053
Surgery Performed (For total 104 patients)	*n* = 55	*n* = 49	0.444
APR	26(47.3%)	22(44.9%)	0.985
LAR	23(41.8%)	22(44.9%)	
Local excision	2(3.6%)	2(4.1%)	
Hartmann	4(7.3%)	3(6.1%)	
Interval between radiotherapy and surgery, days			
<82	27 (49.1%)	23 (46.9%)	0.826
≥82	28 (50.9%)	26 (53.1%)	
LPLD Performed	10(18.2%)	2(4.1%)	**0.025**
R0 Resection	52(94.5%)	46(93.9%)	0.514
Surgery time (range)	199 (60–410) min	212 (30–383) min	–
Smount of bleeding (range)	100 (20–500) ml	100 (5–600) ml	–
Hospital stays after surgery(range)	7 (4–48) days	7(1–105) days	–
pT Stage			
T0	13(23.6%)	12(24.5%)	0.797
T1	4(7.3%)	5(10.2%)	
T2	15(27.3%)	9(18.4%)	
T3	21(38.2%)	22(44.9%)	
T4	2(3.6%)	1(2.0%)	
pN Stage			
N0	38(69.1%)	36(73.5%)	0.541
N1	16(29.1%)	11(22.4%)	
N2	1(1.8%)	2(4.1%)	
pCR	11(20.0%)	10(20.4%)	0.959
TRG Grade			
0	13(23.6%)	11(22.4%)	0.994
1	20(36.4%)	19(38.8%)	
2	21(38.2%)	18(36.7%)	
3	1(1.8%)	1(2.0%)	

LPLN, lateral pelvic lymph node; LPLD, lateral pelvic lymph node dissection; APR, abdominoperineal resection; LAR, low anterior resection; pCR, pathology complete response; TRG, tumor regression grade.

Of the 104 patients who underwent surgery, 98 (94.2%) patients achieved R0 resection, 21 patients (20.2%) achieved pathological complete response (pCR), 74 patients (71.1%) were diagnosed ypN0, and 58 patients (55.8%) were diagnosed with ypT0-2 disease in the final pathology. There were no significant differences in the pathology results between the two groups.

**Figure 1:**
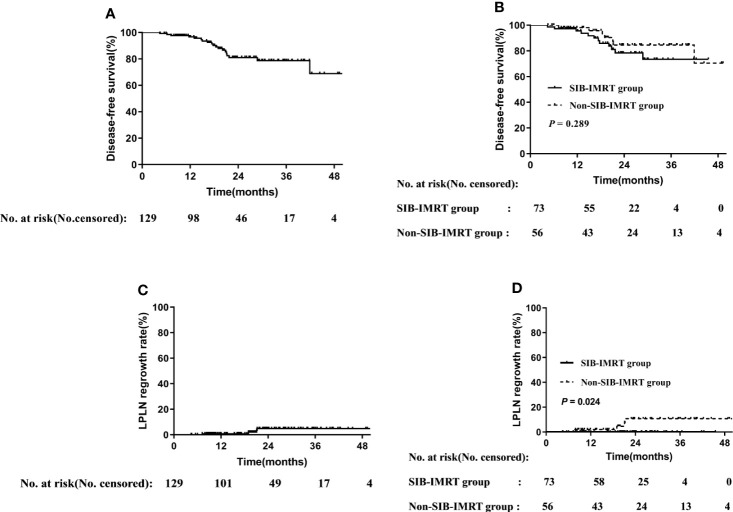
Disease-free survival rate in overall cohort **(A)** and in subgroups of whether receive SIB-IMRT **(B)**. LPLN regrowth rate in the overall cohort **(C)** and in subgroups of whether receive SIB-IMRT **(D)**.

The median follow-up period was 22.6 (range: 4.4–54.9) months, the 2-year DFS in the overall cohort was 80.8% (**Figure 1**), and there was no significant difference in DFS between the two groups (P=0.289).

### LPLN Regrowth

Distant metastasis or refusal of surgery is associated with shorter survival, and thus only patients who achieved cCR or underwent radical surgery were included in the analysis. Of the 129 patients included in the survival analysis, 73 and 56 patients belonged to the SIB-IMRT group and the non-SIB-IMRT group, respectively. The 2-year LPLN regrowth rate in the overall cohort was 4.9% (**Figure 1**). The 2-year LPLN regrowth rate was significantly lower in the SIB-IMRT group than that in the non-SIB-IMRT group (0% vs 10.8%, P=0.024).

**Figure 2:**
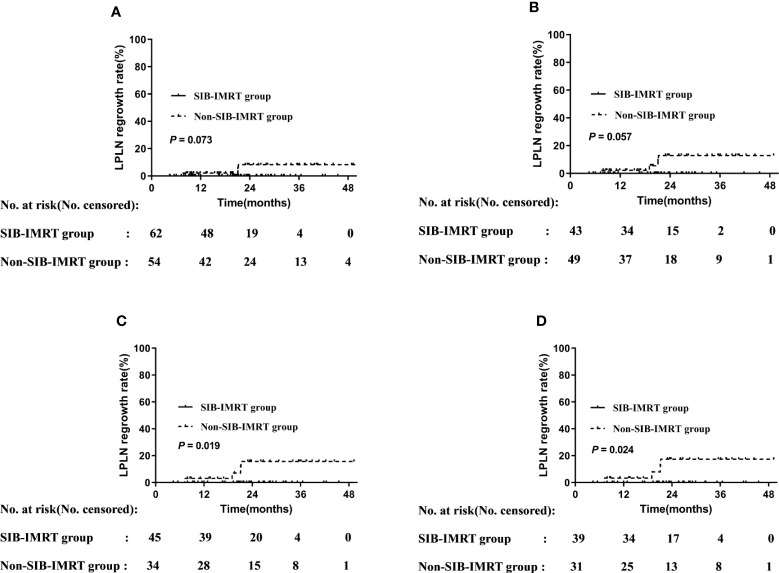
LPLN regrowth rate by subgroup. **(A)** Patients who did not undergo LPLD. **(B)** Patients administered synchronous single-agent chemotherapy. **(C)** Patients whose LPLN short axis was ≥8 mm. **(D)** Patients whose LPLN long axis was ≥10 mm.

Univariable analysis at the subgroup level showed that patients who received LPLD after NCRT, who received synchronous double-agent regimen chemotherapy, whose short axis of LPLNs was <8 mm, and those whose long axis of LPLN was <10 mm did not develop regrowth in the primary LPLN area. Patients in the SIB-IMRT group who did not receive LPLD (0% vs. 8.3%, P=0.073) or who received synchronous single-agent chemotherapy (0% vs. 13.0%, P=0.057) showed lower 2-year regrowth rates of LPLN than did their counterparts in the non-SIB-IMRT group, although the difference was not significant. Meanwhile, patients whose LPLN was ≥8 mm in the short axis (0% vs. 15.9%, P=0.019) or 10 mm in the long axis (0% vs. 17.6%, P=0.024) in the SIB-IMRT group showed better in 2-year regrowth rates of LPLN than did their counterparts in the non-SIB-IMRT group (**Figure 2**).

### Toxicities and Surgical Complications

All patients completed the full-dose radiotherapy plan. Dose reductions of synchronous chemotherapy were needed in 2 patients (1 per group). No toxicity-related death and grade 4 toxicity occurred during chemoradiotherapy, and only 5 patients (3.3%) developed grade III acute toxicity. There were 52 patients (34.4%) who developed grade II acute toxicity.

The most common radiation-induced toxicities were proctitis (16.5%) and leukopenia (12.6%). In total, 104 patients (55 in the SIB-IMRT group and 49 in the non-SIB-IMRT group) were evaluated for complications after surgery. Of them, 18 patients (17.3%) experienced complications after surgery. The details of toxicity and complications are shown in **Table 3**. The χ2 test indicated no significant difference in complications and toxicities between the two groups.

**Table 3:** The toxicity during chemoradiotherapy and complications after surgery.

	SIB-IMRT group (*%*)	Non-SIB-IMRT group (*%*)	*p* value
Any grade II acute toxicity during CRT (For total 151 patients)	25(30.1%)	27(39.1%)	0.217
Leukopenia	10(12.0%)	9(13.2%)	0.827
Proctitis	12(14.5%)	13(19.1%)	0.443
Radiodermatitis	2(2.4%)	4(5.9%)	0.277
Anemia	3(3.6%)	1(1.5%)	0.414
Diarrhea	1(1.2%)	5(7.4%)	0.054
Fatigue	0	1(1.5%)	0.268
Flatulence	1(1.2%)	0	0.364
Postoperative complication (For total 104 patients)	12(21.8%)	6(12.2%)	0.198
Small-bowel obstruction	4(7.3%)	2(4.1%)	0.486
Abdominal wound infections	2(3.6%)	0	0.178
Perineal wound infections	2(3.6%)	0	0.178
Anastomotic leakage	1(1.8%)	0	0.343
Urological	1(1.8%)	3(6.0%)	0.255
Bleeding	1(1.8%)	1(2.0%)	0.934
Electrolyte disturbance	1(1.8%)	0	0.343

CRT, Chemoradiotherapy.

## Discussion

The optimal treatment modality for positive LPLN involvement from LARC remains unclear. The results of this study indicate that SIB-IMRT yields better 2-year regrowth rates of LPLN than non-SIB-IMRT, particularly for LPLNs with a short axis of ≥8 mm or long axis of ≥10 mm. The addition of oxaliplatin in synchronous chemotherapy or LPLD may reduce this difference.

SIB-IMRT has been widely used in recent years, especially for patients who have concerns about the side effects of LPLD. Positive LPLNs are common in distal LARC and are thought to be associated with poor prognosis. Neoadjuvant hemoradiotherapy is recommended for these patients (22). However, some studies indicated that if the primary MRI indicated that the LPLN has a short axis of more than 7 mm, routine chemoradiotherapy may be insufficient. Ryota et al. evaluated 247 patients with enlarged LPLNs who received (chemo)radiotherapy and LPLD, and found that the pretreatment short axis had good discrimination for LPLN metastasis (23). Atsushi et al. investigated 1,216 distal LARC patients and reported that an LPLN short axis of at least 7 mm was associated with a significantly higher rate of lateral local recurrence (15). In our study, the short axis of LPLN in the overall cohort was at least 7 mm. Among the patients who received a total radiotherapy dose of 41.8 Gy, 10.8% experienced LPLN regrowth, indicating that LPLD should be considered for partial patients.

Japanese guidelines recommend LPLD for distal LARC (i.e., lower margin below the peritoneal reflection) (5). The JCOG 0212 trial, the largest randomized controlled, non-inferiority trial, has proven the benefit of LPLD for distal LARC, as it results in a lower local recurrence rate, particularly in patients with lateral pelvic recurrence, compared with single mesorectal extension (24). Long-term data indicated that only stage III patients could benefit from LPLD (25). Meanwhile, LPLD also incurs a longer operation time and results in greater blood loss. However, it does not aggravate urinary dysfunction and male sexual dysfunction, which may be due to the surgeons’ extensive experience and excellent surgery skills (6, 26). One meta-analysis of three studies found a higher prevalence of male sex dysfunction and urinary dysfunction (odds ratio: 3.07, P=0.001), significantly longer operating time, and greater intraoperative blood loss in the LPLD group (7). In the current study, all 12 patients (11.5%) who underwent LPLD after NCRT did not develop LPLN metastasis and sexual and urinary dysfunction during the follow-up.

To avoid the associated negative effects of LPLD, screening for appropriate patients after NCRT is important. Atsushi et al. evaluated 741 patients with cT3/4 rectal cancer within 8 cm from the anal verge, all of whom received NCRT and had restaging MRI. They found that an LPLN short axis of <4 mm was associated with a lower rate of lateral local recurrence (27). Thus, maximizing LPLN shrinkage has become an important aim of neoadjuvant therapy. Some randomized studies have indicated that CapOX-based NCRT does not improve the pCR rate and can even cause a higher acute toxicity rate (28–31). The CAO/ARO/AIO-04 and FORWAC study indicated that double-agent chemoradiotherapy may improve tumor regression (32, 33). Thus, the dosage and period of administration may need further exploration. In our study, the patients who received synchronous double-agent regimen chemotherapy did not develop LPLN regrowth, supporting that appropriate double-agent chemoradiotherapy regimens can benefit select patients.

SIB-IMRT–based chemoradiotherapy achieves better tumor regression in primary rectal cancer (34–36). In this study, we found that if the dose escalation reached 56–60 Gy using SIB-IMRT, none LPLN regrowth occurs during the follow-up. Meanwhile, the toxicities and surgical complications were similar when compared with routine dose chemoradiotherapy. The benefit of oxaliplatin in NCRT for LARC is still unclear. Some randomized studies reported that the additional oxaliplatin during NCRT did not increase the pCR rate and improve the long-term prognosis (28, 30, 31). However, the initial results of the CAO/ARO/AIO-04 trial indicated a higher pCR rate in the fluorouracil and oxaliplatin groups than that in the fluorouracil group. After a median follow-up of 50 months, the fluorouracil and oxaliplatin group also had a significantly higher 3-year DFS (75.9% vs. 71.2%, P=0.03) (37, 38). Similar results were reported by the FORWAC study: the mFOLFOX6 regimen with or without radiation in the neoadjuvant setting for LARC treatment could obtain a higher pCR rate than fluorouracil and leucovorin with radiation (32). These two studies reveal that the appropriate additional oxaliplatin regimen may lead to better tumor regression. In our study, all patients who received synchronous double-agent regimen chemotherapy did not experience LPLN regrowth, suggesting that intensifying the chemotherapy strategy may also be an appropriate treatment for clinically positive LPLN. In the non-SIB-IMRT group, 70.6% of the patients received neoadjuvant chemotherapy in addition to NCRT. Recent studies have indicated that total neoadjuvant chemoradiotherapy may be an effective strategy for high-risk LARC patients (20, 39), warranting further studies on the usefulness of this strategy for patients with clinically positive LPLN.

This study has some limitations owing to its retrospective design. First, there might have been a selection bias because the patients were screened according to the primary MRI findings. Although the criteria included an LPLN short axis of ≥7 mm, this was not indicative of positive LPLN on pathology. Second, only a small percentage of the patients underwent LPLD after NCRT or during the follow-up, and the pathological findings of LPLN are important criteria for evaluating the efficacy of neoadjuvant treatment or diagnosis of LPLN regrowth. Third, the dose delivered in the non-IMRT-SIB group was slightly lower than the standard dose usually delivered in preoperative CRT for rectal cancer. Meanwhile, in our previous studies to explore the effectiveness of this NCRT plan, the ypN negative rate and long-term prognosis were not inferior to those in standard radiotherapy (17, 40). Fourth, after NCRT, five patients who underwent surgery at other hospitals were excluded because we could not determine the quality of surgery. Further, due to the COVID-19 pandemic, radiological evaluation was delayed in the partial patients. Lastly, our study was limited by a single center design and a relatively short duration of follow-up. Further studies are needed to investigate the usefulness of intensifying the chemotherapy strategy, and we are currently planning a multicenter prospective study to validate our results.

In conclusion, SIB-IMRT–based chemoradiotherapy could better eliminate clinically positive LPLNs, particularly for patients who undergo traditional NCRT and have larger LPLN. Further, it yields similar incidence rates of toxicity and surgical complications to those of non-SIB-IMRT.

## Data Availability Statement

The raw data supporting the conclusions of this article will be made available by the authors, without undue reservation.

## Ethics Statement

The studies involving human participants were reviewed and approved by the institutional review board of Ethics Committee of Beijing Cancer Hospital. The patients/participants provided their written informed consent to participate in this study.

## Author Contributions

Conception and design of study: WW and YC. Acquisition of data/drafting the manuscript: SL, YZ, and YY. Analysis and/or interpretation of data: XZ, JG, HT, ZW, TS, LW, HW, YL, and AW. All authors contributed to the article and approved the submitted version.

## Funding

(1) Science Foundation of Peking University Cancer Hospital No. 18-03; (2) Beijing Municipal Science &Technology Commission No. Z181100001718192; (3) Beijing Natural Science Foundation No. 7182028; (4) Capital’s Funds for Health Improvement and Research (2020-2-1027); (5) Sailing Project - Clinical Technology Innovation Project (No. XMLX201842)

## Conflict of Interest

The authors declare that the research was conducted in the absence of any commercial or financial relationships that could be construed as a potential conflict of interest.
