# Corrigendum: Simultaneous Integrated Boost Intensity-Modulated Radiation Therapy Can Benefit the Locally Advanced Rectal Cancer Patients with Clinically Positive Lateral Pelvic Lymph Node

**DOI:** 10.3389/fonc.2021.790945

**Published:** 2021-12-14

**Authors:** Shuai Li, Yangzi Zhang, Yang Yu, Xianggao Zhu, Jianhao Geng, Huajing Teng, Zhilong Wang, Tingting Sun, Lin Wang, Hongzhi Wang, Yongheng Li, Aiwen Wu, Yong Cai, Weihu Wang

**Affiliations:** ^1^ Department of Radiation Oncology, Key Laboratory of Carcinogenesis and Translational Research (Ministry of Education/Beijing), Peking University Cancer Hospital and Institute, Beijing, China; ^2^ Department of Gastrointestinal Surgery, Key Laboratory of Carcinogenesis and Translational Research (Ministry of Education), Peking University Cancer Hospital & Institute, Beijing, China; ^3^ Department of Radiology, Key Laboratory of Carcinogenesis and Translational Research (Ministry of Education), Peking University Cancer Hospital & Institute, Beijing, China

**Keywords:** simultaneous integrated boost intensity-modulated radiation therapy, neoadjuvant chemoradiotherapy, lateral pelvic lymph node, local advanced rectal cancer, regrowth rate, disease-free survival

In the original article, there was a mistake in the legend for **Figure 1** and as published.

**Figure 1:**
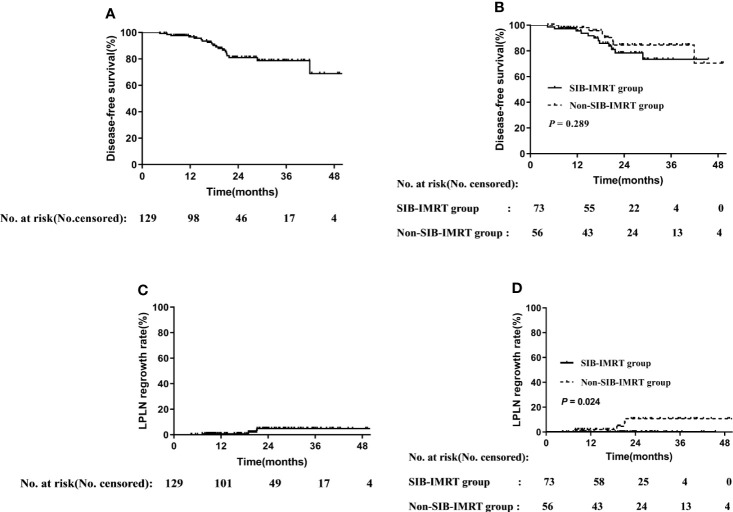
Disease-free survival rate in overall cohort **(A)** and in subgroups of whether receive SIB-IMRT **(B)**. LPLN regrowth rate in the overall cohort **(C)** and in subgroups of whether receive SIB-IMRT **(D)**.

The **Figure 1D** should be an increasing curve, consistent with **Figure 1C**. The data was correct, and the error occurred when choosing the curve. The correct legend appears below.

In the original article, there was a mistake in the legend for **Figure 2** as published. The **Figure 2** is same as **Figure 1**, which is inconsistent with the manuscript at the time of submission. The correct legend appears below.

**Figure 2:**
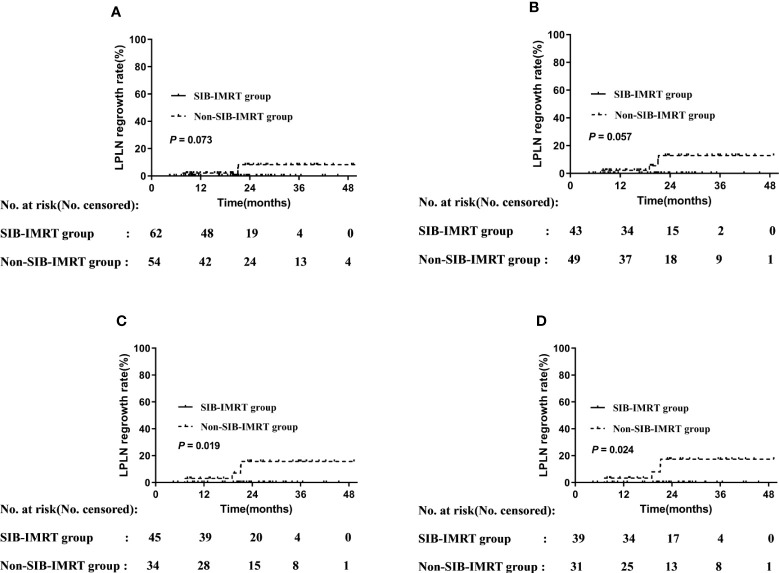
LPLN regrowth rate by subgroup. **(A)** Patients who did not undergo LPLD. **(B)** Patients administered synchronous single-agent chemotherapy. **(C)** Patients whose LPLN short axis was ≥8 mm. **(D)** Patients whose LPLN long axis was ≥10 mm.

The authors apologize for these errors and state that this does not change the scientific conclusions of the article in any way. The original article has been updated.

## Publisher’s Note

All claims expressed in this article are solely those of the authors and do not necessarily represent those of their affiliated organizations, or those of the publisher, the editors and the reviewers. Any product that may be evaluated in this article, or claim that may be made by its manufacturer, is not guaranteed or endorsed by the publisher.

