# Supplementary figures and images for: Simultaneous Integrated Boost Intensity-Modulated Radiation Therapy Can Benefit the Locally Advanced Rectal Cancer Patients With Clinically Positive Lateral Pelvic Lymph Node
Source: Front Oncol. 2021 Feb 22;10:627572. doi: 10.3389/fonc.2020.627572 (PMC7937798; doi:10.3389/fonc.2020.627572)

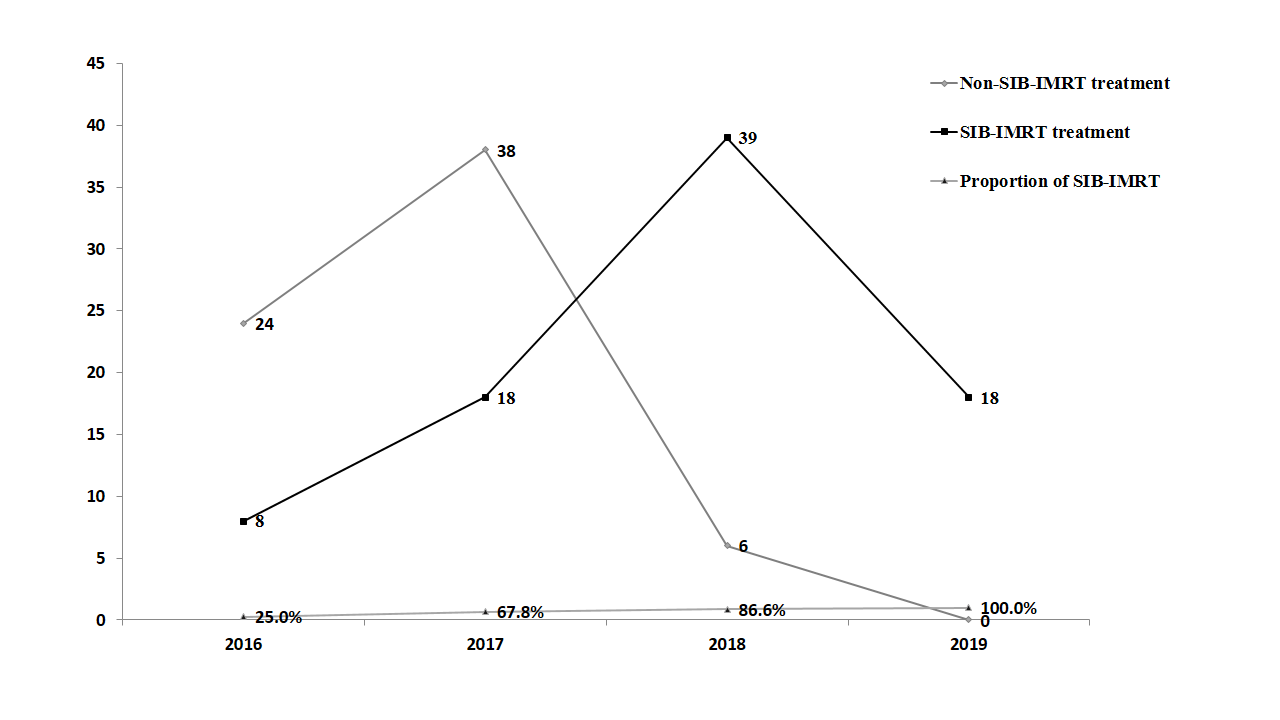

Supplement: Supplementary file 1 [file Image_1.tif]
